# Somatic BRCA Mutation in a Cholangiocarcinoma Patient for HBOC Syndrome Detection

**DOI:** 10.3389/fonc.2020.01292

**Published:** 2020-08-12

**Authors:** Angelo Virgilio Paradiso, Margherita Patruno, Maria Digennaro, Stefania Tommasi, Brunella Pilato, Antonella Argentiero, Oronzo Brunetti, Nicola Silvestris

**Affiliations:** ^1^Experimental Oncology—Center for the Study of Hereditary Cancers, IRCCS-Istituto Tumori “Giovanni Paolo II”, Bari, Italy; ^2^Scientific Direction, IRCCS-Istituto Tumori “Giovanni Paolo II”, Bari, Italy; ^3^Molecular and Pharmacogenetics Diagnostic Laboratory, IRCCS-Istituto Tumori “Giovanni Paolo II”, Bari, Italy; ^4^Medical Oncology Unit, IRCCS-Istituto Tumori “Giovanni Paolo II”, Bari, Italy; ^5^Department of Biomedical Sciences and Human Oncology, University of Bari “Aldo Moro”, Bari, Italy

**Keywords:** BRCA1, BRCA2, biliary tract cancers, hereditary BRCA cancer, somatic mutations

## Abstract

BRCA-associated hereditary breast and ovarian cancer syndrome (HBOC) is characterized by an increased risk of developing other malignancies including cholangiocarcinoma (CCA). Somatic BRCA mutations have been reported in CCA, but they have yet to be utilized in a proband case to identify HBOC in families. Two healthy daughters of a deceased female patient who had had metachronous breast cancer and CCA received genetic counseling to assess their cancer risk. Somatic *BRCA1/2* mutation analysis was performed by next-generation sequencing on the DNA extracted from a formalin-fixed, paraffin-embedded CCA biopsy specimen of their mother. A pathogenic variant was identified (c.6468_6469delTC in a BRCA2 gene mutation). Germline BRCA mutation analysis of the two daughters detected the same pathogenic variant in one of them. For the first time, a CCA somatic BRCA mutation has been used to identify a family with HBOC.

## Introduction

*BRCA1-* and *BRCA2-*associated hereditary breast and ovarian cancer (HBOC) syndrome is characterized by an increased risk of breast cancer and gynecological cancers in most cases (1). When this syndrome is suspected, investigation of germline BRCA mutations based on specific risk factors is required (2). Hereditary breast and ovarian cancer syndrome has also been associated with an increased risk of other cancers such as prostate cancer, pancreatic ductal adenocarcinoma, and melanoma (3). The use of somatic *BRCA* information to select patients to be treated with specific molecular targeting approaches has been also suggested (2).

BRCA mutations were detected in 3.6% of biliary tract malignancies examined in a recent study (4). However, to date, no specific indications regarding genetic testing in this subset of patients have been provided.

In this article, we report the results of genetic counseling and testing in two healthy daughters of a deceased female patient who had had metachronous breast cancer at 39 years of age and a cholangiocarcinoma (CCA) at 65 years. Because germinal DNA from the index case was unavailable, somatic *BRCA1/2* mutation analysis was performed on the DNA extracted from the formalin-fixed, paraffin-embedded CCA biopsy material. A BRCA2 mutation was detected, and one of the two daughters tested positive for the same mutation, thus confirming HBOC syndrome in the family. To our knowledge, this is the first time CCA tissue was used for this purpose.

## Patients and Methods

The clinical history of a deceased female patient and that of her two healthy daughters were obtained at the *IRCCS Istituto Tumori “Giovanni Paolo II*” (Cancer Institute) in Bari, Italy, where the patient had been treated 10 years earlier. Written informed consent was obtained from the two daughters and approved by the Ethical Committee (protocol no. 700/18) of the *IRCCS Istituto Tumori “Giovanni Paolo II*” in Bari, Italy. Genetic counseling was managed by the Center for the Study of Hereditary Cancers of the *Istituto Tumori*, and all the laboratory tests were performed in the Molecular and Pharmacogenetics Diagnostic Laboratory of the same institution.

Genetic analysis was carried out on the DNA extracted from the deceased patient's tumor tissue using the QiAmp DNA FFPE kit (Qiagen, Valencia, CA, USA) and following the manufacturer's instructions. Mutation analysis was performed with the Oncomine BRCA1-2 Assay (Thermo Fisher Scientific, Carlsbad, CA, USA). This panel generates 265 amplicons, and it was designed for 100% amplicon coverage of all targeted coding exons and exon–intron boundaries. The DNA was 20 ng, and all the libraries were constructed following the manufacturer's recommendations. After quantification, all the libraries were diluted to a final concentration of 100 pM and combined equimolarly. Library stock dilutions were used for the preparation of enriched, template-positive, ion sphere particles. Automated protocols were run on the Ion One Touch 2 System and Ion One Touch ES Instrument (Thermo Fisher Scientific) as described elsewhere (5). All the data were analyzed using the Torrent Server Suite, and variants were called with the Variant Caller Plugin using parameter sets and hotspot files as previously described. Given that the analysis was performed on a tissue sample, special care was taken to check that all the readings generated had a coverage of 500X and that all the variants had an allele frequency >10% (6). All the variants identified were confirmed by Sanger Sequencing as previously described (5, 7).

## Results

Two healthy sisters with a family history of cancer received genetic counseling at our institution in order to assess their personal cancer risk. Their mother had been diagnosed with breast cancer at the age of 39 years and had no specific breast cancer–related recurrence. At the age of 65 years, despite her healthy lifestyle and no other risk factors (smoking, alcohol use, obesity), radiodiagnostic tests showed multiple liver lesions during a preoperative evaluation for a uterine prolapse. Upon liver biopsy, hepatic localizations of moderately differentiated adenocarcinoma with desmoplastic stroma were diagnosed. The morphological and immunohistochemical profile were consistent with a diagnosis of a CCA (CK ++; CK19 ++; CEA++; EMA + + +; CAM 5.2 + + +; TTF1, CDX2, CK20, ER, and PgR: negative).

**Figure 1:**
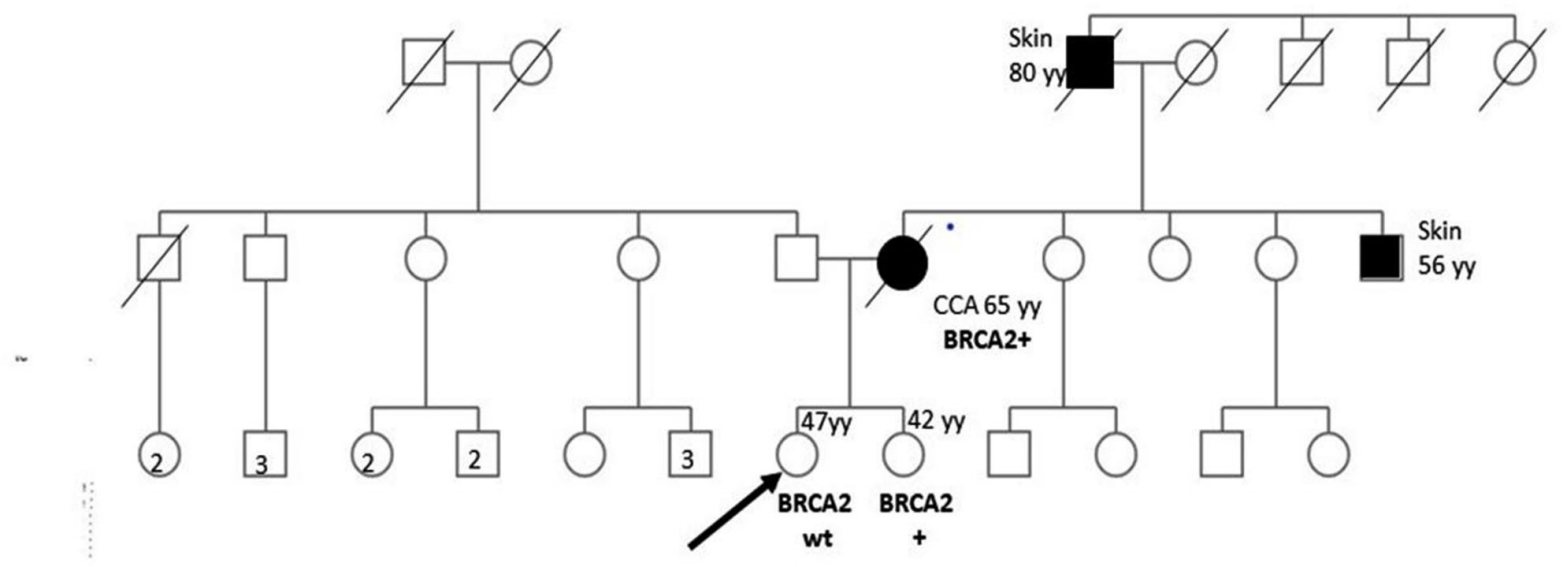
Genealogical tree of the family with HBOC syndrome. CCA, cholangiocarcinoma; wt, wild type; yy, years; HR, homologous recombination.

Figure 1 shows proband's maternal and paternal genealogical tree.

Because of the presence of two metachronous cancers, HBOC syndrome was hypothesized, and the possibility of performing *BRCA1/2* mutation analysis on a biopsy sample taken from their mother was discussed with both daughters.

*BRCA1/2* next-generation sequencing (NGS) was performed on the DNA extracted from the patient's formalin-fixed, paraffin-embedded CCA biopsy material. The only pathogenic variant identified was *c.6468_6469delTC; p.Gln2157Ilefs (rs80359596)* in the exon 11 of the *BRCA2* gene with a variant allele frequency of >40%. We looked for the same variant in the DNA extracted from the peripheral blood lymphocytes (PBLs) of both sisters assuming that the identified variant could be germinal. The specific variant was found in one of the sisters, whereas no mutations were found in the other (Figure 1). In such a case, cancer screening practices may be modified and adapted to the increased cancer risk of the BRCA mutated woman.

## Discussion

We have described a case in which HBOC syndrome was identified utilizing CCA tissue from a deceased proband, a female patient who had also had breast cancer at 39 years of age. Detailed information concerning her breast cancer was unavailable because of the long time that had elapsed since her death.

BRCA testing is ordinarily performed on germline DNA except in cases when this biological material is unavailable, as in ours. In such instances, somatic *BRCA1/2* NGS analysis has been performed on DNA extracted from formalin-fixed, paraffin-embedded biopsy material (5). In our case, somatic BRCA mutation analysis was performed on the proband's paraffin-embedded CCA biopsy specimen, and a BRCA germline mutation test was carried out in her daughters. The same pathogenic variant *(c.6468_6469delTC* in the *BRCA2* gene) was found in the CCA biopsy specimen and in the DNA extracted from the PBLs of one daughter. To the best of our knowledge, this report regarding the use of BRCA assessment in CCA to diagnose HBOC syndrome is the first of its kind. A previous study (5) described the possibility of performing *BRCA1/2* analysis on ovarian and breast cancer specimens, but this is the first time genetic DNA testing of a CCA biopsy specimen has been used to reveal a cancer predisposition syndrome. Identification of an individual harboring a BRCA mutation by this means should encourage clinicians to address enhancing cancer screening provisions in CCA patients (8).

Our experience also points to the possibility of seeking “actionable” BRCA mutations in CCA that may be potential druggable targets. The findings from our case report are in agreement with results from a previous large series of CCA patients (4, 9), which showed that BRCA mutations in CCA are not a rare event. Hence, *BRCA1/2* mutations in CCA are not to be considered only as biomarkers of hereditary cancer risk but also as having potential therapeutic implications. Cholangiocarcinoma is a challenging disease in terms of both diagnosis and treatment because of its poor survival and chemoresistance (10). PARP (poly(ADP-ribose) polymerase) inhibitors demonstrated activity in *BRCA1/2* mutated CCA patients. In a recent retrospective analysis of 18 patients with *BRCA1/2* mutated CCA (6 patients with extrahepatic CCA and 12 with intrahepatic CCA), four patients were treated with PARP-i, with one achieving a sustained disease response and a progression-free survival of 42.6 months (11). Median overall survivals were 40.3 months [95% confidence interval (CI), 6.73–108.15 months] and 25 months (95% CI, 15.23–40.57 months) for stages I/II and III/IV patients, respectively.

In conclusion, thanks to the NGS technology, we were able to identify somatic mutations even from small amounts of tumor tissue. More emphasis should be given to somatic BRCA analysis in CCA given the frequency of mutations in these genes and the implications of such mutations for prevention and therapy in clinical practice.

## Ethics Statement

Written informed consent was obtained for the publication of any potentially identifiable images or data included in this article.

## Author Contributions

AP, MP, MD, ST, and BP conceived and planned the experiments, carried out the experiments. AP, MP, BP, OB, and NS contributed to sample preparation and interpretation of the results. AP, MP, ST, BP, OB, and NS took the lead in writing the manuscript. AP, OB, and NS revised the clinical history. All authors provided critical feedback, helped shape the research and analysis, and revised the manuscript.

## Conflict of Interest

The authors declare that the research was conducted in the absence of any commercial or financial relationships that could be construed as a potential conflict of interest.
